# Adjuvant chemotherapy in rectal cancer patients who achieved a pathological complete response after preoperative chemoradiotherapy: a systematic review and meta-analysis

**DOI:** 10.1038/s41598-019-46457-5

**Published:** 2019-07-10

**Authors:** Yu Jin Lim, Youngkyong Kim, Moonkyoo Kong

**Affiliations:** Department of Radiation Oncology, Kyung Hee University Medical Center, Kyung Hee University School of Medicine, Seoul, Republic of Korea

**Keywords:** Chemotherapy, Radiotherapy, Rectal cancer

## Abstract

This study evaluated the prognostic impact of ACT in patients who achieved a pathological complete response (pCR). Articles published from January 1990 to September 2018 were searched in EMBASE, PubMed, Ovid, Web of Science, and Cochrane Library. Hazard ratios (HRs) and 95% confidence intervals (CIs) of overall survival (OS) were extracted. Thirteen observational studies were included. There were four National Cancer Database studies with overlapping study periods, thus individual pooled analyses of four different datasets were conducted (n = 3,182, 3,330, 3,575, and 4,739 for pooled analysis sets including Dossa *et al*., Polanco *et al*., Xu *et al*., and Shahab *et al*., respectively). Although a trend toward improved OS with ACT was observed, statistical significance was not proven (*P* = 0.09, *P* = 0.03, *P = *0.12, and *P* = 0.10, respectively). When we performed a stratified analysis comparing the results from single institution and multicenter studies, there was no significant prognostic benefit of ACT. Publication bias was not observed. Routine use of ACT in patients with a pCR could not be warranted from the present meta-analysis. Further study of individual patient data from randomized trials is needed to clarify the role of ACT.

## Introduction

Colorectal cancer is the third most common type of malignancy and ~43,030 rectal cancer cases are newly diagnosed annually in the United States^1^. The widespread use of total mesorectal excision combined with multimodality treatments has improved oncologic outcomes, but the rates of distant metastasis after long-term follow-up remains high, at 20–35%^2,3^.

Following a landmark German trial^4^, recent National Comprehensive Cancer Network (NCCN) and European guidelines have recommended preoperative chemoradiotherapy (CRT) plus surgical resection as the standard treatment for locally advanced rectal cancer^5,6^. After completion of transabdominal surgery, adjuvant chemotherapy (ACT) has generally been recommended^5^. However, the clinical efficacy of the postoperative strategy has been questioned in the era of neoadjuvant CRT. The rationale for ACT has been extrapolated from the results of colon cancer cases, as well as an earlier meta-analysis that reported improved prognosis with ACT mainly in the context of upfront surgery followed by postoperative treatment^7^. According to the 5-year results of the European Organization for Research and Treatment of Cancer (EORTC) 22921 trial, the use of ACT improved disease-free survival (DFS) and overall survival (OS) in cases with intermediate down-staging due to CRT^8^. However, a long-term analysis of 10-year data failed to show similar survival differences^3^. Given the lack of randomized evidence, the indications for ACT in rectal cancer clinics are controversial^9–13^.

A pathological complete response (pCR), coded ypT0N0, is importantly associated with favorable prognosis in rectal cancer. Since a pCR is seen in only a small percentage (10–20%) of rectal cancer patients^14,15^, no prospective study designed to assess the survival benefits of ACT in these individuals has been conducted. Here, we performed a systematic review and meta-analysis to evaluate the impact of ACT on the survival of rectal cancer patients who achieved ypT0N0 status. The OS outcomes of two groups, with and without the postoperative use of chemotherapy, were compared. Given the paucity of randomized trials appertaining to pCR status, the present pooled analysis provides clinical insights into the role of ACT in patients who achieved remarkable tumor eradication following CRT.

## Methods and Materials

### Literature search strategy

A systematic search of electronic databases was conducted to identify studies that analyzed OS in locally advanced rectal cancer patients treated with ACT. The search process followed the Preferred Reporting Items for Systematic Reviews and Meta-analyses (PRISMA) guidelines. The outcomes of the present meta-analysis were reported according to the Meta-analysis of Observational Studies in Epidemiology (MOOSE) criteria (Supplementary Materials Appendix 1)^16^. Published articles that compared ACT and non-ACT groups of rectal cancer patients treated with neoadjuvant CRT plus surgery from January 1990 to September 2018 were identified by searching the EMBASE, PubMed, Ovid, Web of Science, and Cochrane Library databases. Hand searches were also performed to identify other potentially eligible studies, but no additional studies were added. The following keywords, synonyms, and combinations thereof were used as search terms: [rectum] OR [rectal]; [cancer] OR [carcinoma]; [postoperative] OR [adjuvant]; [chemotherapy]; [preoperative] OR [neoadjuvant]; [chemoradiotherapy] OR [chemoradiation] (Supplementary Materials Appendix 2). No restriction on study design was considered.

### Study selection

Two independent authors (YJL and MK) performed the searches and assessed study eligibility. Studies comparing OS between postoperative ACT (intervention group) and observation alone (comparator group) in pCR patients were selected. Exclusion criteria applied during the selection process were as follows: (1) conference abstracts; (2) unstructured papers, such as editorials, comments, and letters; (3) case reports and review articles; (4) studies not reporting the survival outcome of pCR patients; (5) lack of information regarding a comparator group; and (6) insufficient OS data to extract hazard ratios (HRs) and 95% confidence intervals (CIs). The name of the institution or database included in the final set of eligible studies was reviewed. When multiple studies were based on the same data, the one with a longest-duration study period and the largest number of patients was selected. The study selection process was verified independently by a third investigator (YK).

### Data extraction

The data were extracted independently by two authors (YJL and MK). When discrepancies occurred, the authors discussed to reach a consensus. Some authors of potentially eligible studies were contacted via e-mail to request required data, and one study replied^17^.

### Risk of bias assessment

A risk of bias assessment was conducted independently by two authors (YJL and MK). Since all of the included studies were non-randomized observational studies, the Risk of Bias Assessment tool for Nonrandomized Studies (RoBANS) was used to assess the following six domains: the selection of participants; confounding variables; intervention measurement; blinding of the outcome assessment; incomplete outcome data; and selective outcome reporting^18^. Regarding potential discrepancies between the two authors, a consensus was obtained after further review and discussion. In addition, a third investigator (YK) verified the results.

### Statistical analysis

The primary outcome of interest was OS. The HRs and 95% CIs from each study were either extracted directly from original papers or calculated using Kaplan-Meier OS curves based on the method of Tierney *et al*.^19^. HRs were calculated using a random-effects model with the inverse variance method. Cochrane Q tests and the I^2^ index were used to evaluate heterogeneity. Funnel plots with Egger’s regression tests were used to examine publication bias. An additional stratified analysis comparing results from single institution and multicenter studies was performed. RevMan software (ver. 5.3; Cochrane Collaboration, Copenhagen, Denmark) was used for all pooled analyses.

## Results

### Study characteristics

**Figure 1:**
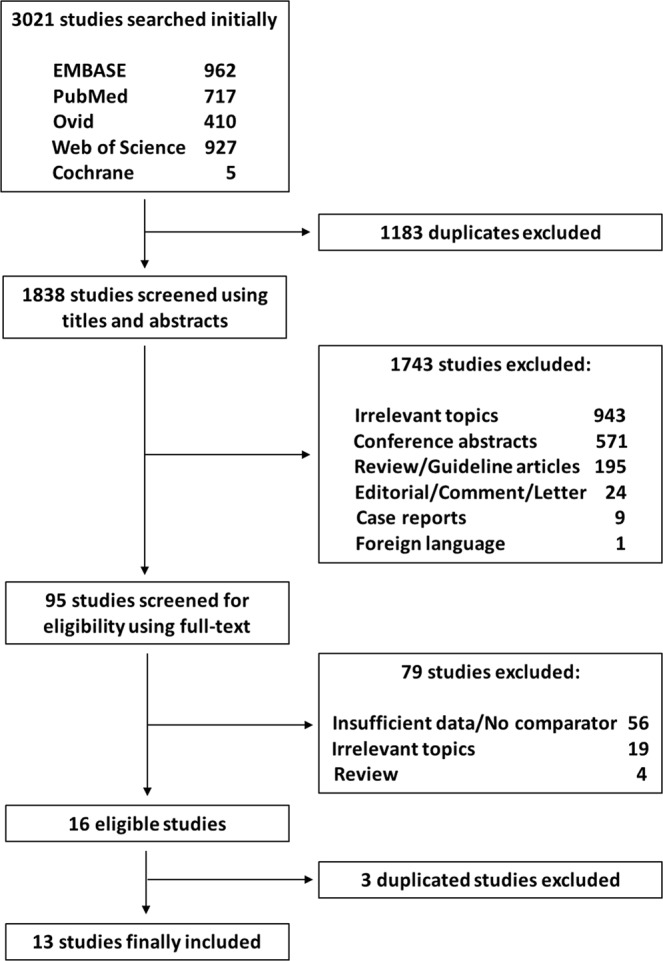
Flowchart of the study selection process.

A flowchart of the study selection process is shown in Fig. 1. Among the 3,021 studies identified initially, the titles and abstracts of 1,838 studies were screened. Irrelevant articles, in terms of their structure or content, were excluded, and 95 manuscripts were reviewed. Further screening then identified 16 studies with sufficient OS data on a potentially eligible pCR population. Of these, three studies that had duplicate data (i.e., from the same institution) but shorter follow-ups, were excluded. Since four different National Cancer Database (NCDB)-based studies overlapped in terms of study period, we included all studies and separately conducted pooled analyses on each of them. A total of 13 studies were eligible^17,20–31^.

Table 1 lists the characteristics of included studies. We investigated five single-institution^17,20–23^ and four multicenter studies^24–27^, and four NCDB analyses (n = 3,182, 3,330, 3,575, and 4,739 for pooled analysis sets I, II, III, and IV defined as datasets including Dossa *et al*., Polanco *et al*., Xu *et al*., and Shahab *et al*., respectively)^28–31^. The time interval between CRT and surgery was typically 1–3 months, and the total radiation dose was 45–50 Gy. Although the details of the chemotherapy regimens used were not included in the NCDB-based studies, most other studies reported fluoropyrimidine-based regimens. Most patients underwent total mesorectal excision. The summarized results regarding risk of bias revealed that the overall quality of the included studies was relatively high (Table 2).

**Table 1:** Characteristics of 13 studies for the present pooled analysis.

	Dossa^a^	Polanco^a^	Song	Xu^a^	Shahab^a^	Kuan	Lorenzon	Gamaleldin	Tay	Kim	Lichthardt	Maas	Geva
Source of data	NCDB	NCDB	Seoul Univ Bundang	NCDB	NCDB	TCRD	Multi-institutional	Cleveland	ACCORD	Yonsei	Wuerzburg	Multi-institutional	Tel Aviv
Country	USA	USA	Korea	USA	USA	Taiwan	Italy/Spain	USA	Australia	Korea	Germany	Multi-national	Israel
Year of publication	2018	2018	2018	2017	2017	2017	2017	2017	2017	2017	2017	2015	2014
Study period	2006–2012	2006–2012	2004–2015	2006–2011	2006–2013	2007–2013	2005–2015	2000–2012	2003–2014	2001–2013	1992–2013	Variable	2001–2013
Data adjustment	Yes	Yes	No	Yes	Yes	Yes	No	No	No	Yes	No	Yes	No
No. of pCR patients (ACT/No ACT)	1334 (667/667)	1482 (741/741)	50 (43/7)	1727 (484/1243)	2891 (789/2102)	259^b^ (114/145)	232 (77/155)	130^c^ (47/83)	126 (97/29)	77 (37/40)	24 (9/15)	898 (290/608)	52 (35/17)
Proportion of CRT(%)	100	100	100	100	100	100	NA^d^	100	100	100	NA^e^	100	100
CRT-Op interval	5–12 weeks for 84%	NA	Median 46.5 weeks (36–76)	NA	NA	<6/6–8/>8 weeks for 21%/46%/33%	Median 9 weeks (3–25)	NA	Median 6.9 weeks	4–8 weeks	NA	Generally 6–8 weeks	5.7–13.6 weeks
RT dose	45–54 Gy for 76%	NA	50.4 Gy	NA	NA	40–51 Gy for 93%	Median 50.4 Gy (50.4–56) for long-course CRT, short-course RT (9%) included	Median 50.4 Gy	Median 50 Gy	45–50.4 Gy	NA	45–50.4 Gy	Median 50.4 Gy (45–50.4)
ACT regimen	NA	NA	5-FU based (including FL, capecita-bine, FOLFOX)	NA	NA	5-FU-based (including FL, tegafur or capecita-bine)	Oral/i.v. fluoro-pyrimidine	5-FU or FL^f^	Fluoro-pyrimidine^g^	FL, capecita-bine	5-FU, capecita-bine, FOLFOX/ FOLFIRI	Fluoro-pyrimidine-based	5-FU, capecita-bine
Types of surgery	NA^h^	Partial/total proc-tectomy for 61%/24%	LAR or U-LAR	NA	Partial/total proc-tectomy for 71%/28%	LAR/APR for 72%/12%	LAR/APR for 75%/16%	Total mesorectal excision	NA	LAR/APR	Total mesorectal excision	LAR/APR for 76%/22%	LAR/APR for 69%/31%

^a^Each of these studies of NCDB was included in the different sets of pooled analyses respectively.

^b^Twenty-two (6 and 16 with and without ACT, respectively) death events were reported.

^c^Six (3 and 3 with and without ACT, respectively) death events were reported.

^d^The proportion of CRT in the entire study population was 90.8%.

^e^The proportion of CRT in the entire study population was 59.7%.

^f^The time to closure of ileostomy was longer in the ACT group (vs. non-ACT), mean 7.1 ± 8.6 months vs. 4.3 ± 3.5 months.

^g^Sixty of 452 patients (including both pCR and non-pCR) stopped treatment due to toxicity, such as, diarrhea, nausea, and vomiting.

^h^Cases of nonresectional ablative procedures or local excision were excluded.

NCDB: National Cancer Database; TCRD: Taiwan Cancer Registry Database; ACCORD: Australian Comprehensive Cancer Outcomes and Research Database; pCR: pathologic complete response; ACT: adjuvant chemotherapy; CRT: chemoradiotherapy; NA: not available; Op: operation; RT: radiotherapy; 5-FU: 5-fluorouracil; FL: 5-fluorouracil/leucovorin; FOLFOX: folinic acid/fluorouracil/oxaliplatin; FOLFIRI: folinic acid/fluorouracil/irinotecan; LAR: low anterior resection; APR: abdominoperineal resection.

**Table 2:** A summary of risk of bias assessment using the Risk of Bias Assessment Tool for Non-randomized Studies (RoBANS).

References (publication year)	Selection		Performance	Detection	Attrition	Reporting
	Selection of participants	Confounding variables	Measurement of exposure	Blinding of outcome assessments	Incomplete outcome data	Selective outcome reporting
Song *et al*.^17^	Low	Low	Low	Low	Low	Unclear
Gamaleldin *et al*.^20^	Low	High	Low	Low	Unclear	Low
Kim *et al*.^21^	Low	Low	Low	Low	Low	Unclear
Lichthardt *et al*. ^22^	Low	High	Low	Low	Unclear	Low
Geva *et al*.^23^	Low	High	Low	Low	Low	Low
Kuan *et al*.^24^	Low	Low	Low	Low	Low	Low
Lorenzon *et al*.^25^	High	High	Low	Low	Low	Low
Tay *et al*.^26^	Low	High	Low	Low	Unclear	Low
Maas *et al*.^27^	High	Low	Low	Low	Low	Low
Dossa *et al*.^28^	Unclear	Low	Low	Low	Unclear	Low
Polanco *et al*.^29^	Unclear	Low	Low	Low	Low	Low
Xu *et al*.^30^	Unclear	Low	Low	Low	Unclear	Unclear
Shahab *et al*.^31^	Unclear	Low	Low	Low	Unclear	Low

### Comparison of OS with and without ACT

**Figure 2:**
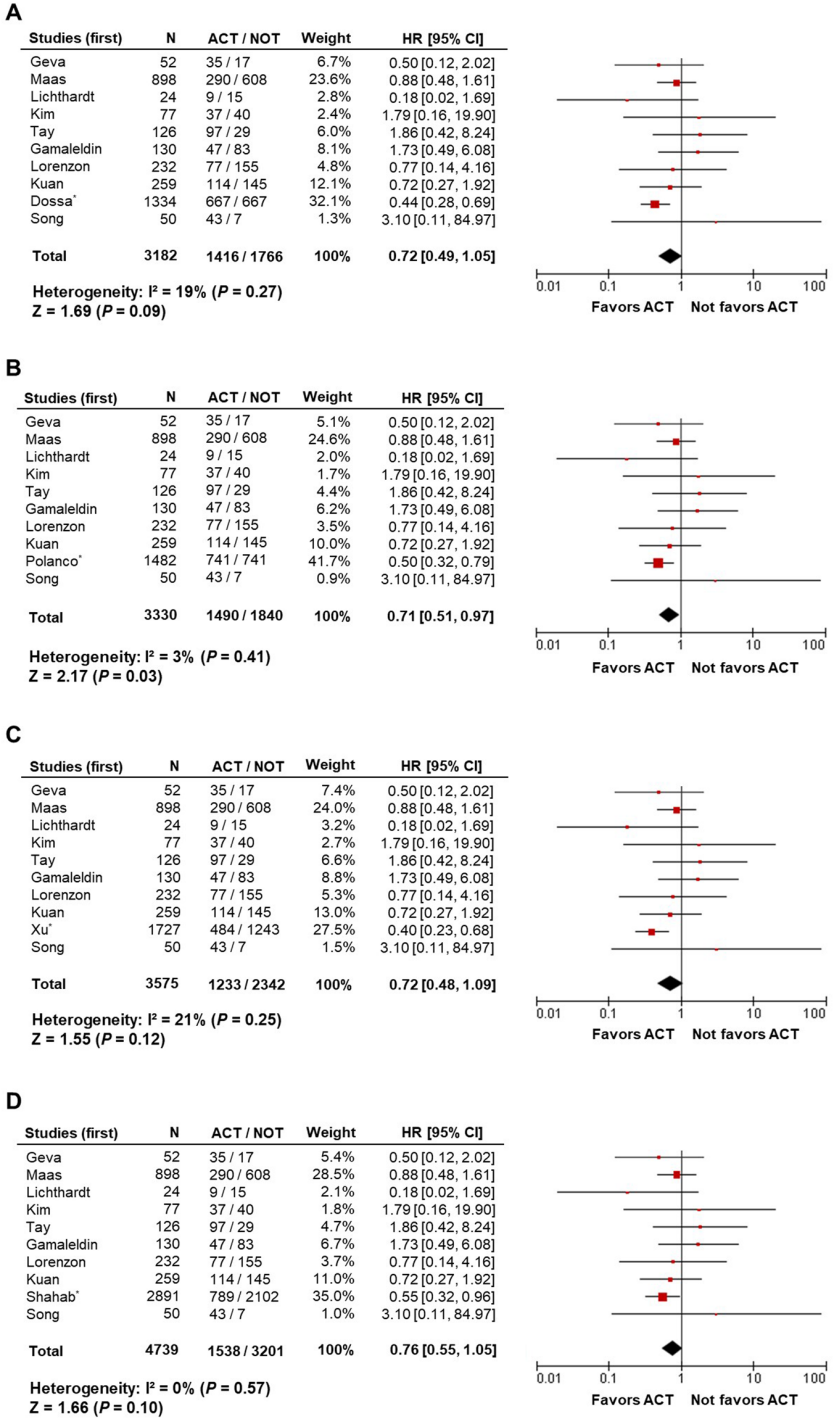
Overall survival comparing adjuvant chemotherapy and observation alone in patients with a pathologic complete response. Forest plots of pooled analysis sets (**A**) I, (**B**) II, (**C**) III, and (**D**) IV, including four different NCDB-based studies of Dossa *et al*., Polanco *et al*., Xu *et al*., and Shahab *et al*., respectively. HR: hazard ratio; CI: confidence interval; ACT: adjuvant chemotherapy. ^*^Four NCDB-based studies.

Individual forest plots of OS data were generated for the four different NCDB-based studies (pooled analysis sets I, II, III, and IV, Fig. 2A–D). Although a trend toward better OS with ACT was observed, statistical significance was not consistent in the different sets of analyses (HR 0.72, 95% CI 0.49–1.05, *P* = 0.09; HR 0.71, 95% CI 0.51–0.97, *P* = 0.03; HR 0.72, 95% CI 0.48–1.09, *P* = 0.12; and HR 0.76, 95% CI 0.55–1.05, *P* = 0.10 for pooled analysis sets I, II, III, and IV, respectively). No significant heterogeneity was observed (I^2^ = 19%, *P* = 0.27; I^2^ = 3%, *P* = 0.41; I^2^ = 21%, *P* = 0.25; and I^2^ = 0%, *P* = 0.57 for pooled analysis sets I, II, III, and IV, respectively).

### Stratified analysis

**Figure 3:**
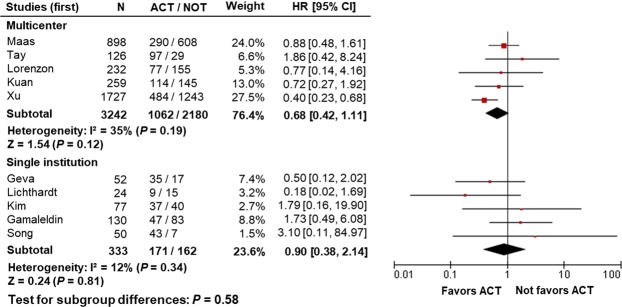
Representative results of stratified analysis comparing results from single institution and multicenter studies using the pooled analysis set III, including the NCDB-based study of Xu *et al*. HR: hazard ratio; CI: confidence interval; ACT: adjuvant chemotherapy.

To compare the results from single institution and multicenter studies, a stratified analysis was performed. Using the pooled analysis set III, which included the study of Xu *et al*., ACT did not result in significant survival benefits in either multicenter (HR 0.68, 95% CI 0.42–1.11, *P* = 0.12) or single-institution (HR 0.90, 95% CI 0.38–2.14, *P* = 0.81) studies (Fig. 3). Other subgroup results based on pooled analysis sets I, II, and IV also showed comparable results (data not shown).

### Publication bias

There was no publication bias in the overall pooled results (*P* = 0.167, *P* = 0.210, *P* = 0.225, and *P* = 0.365 for pooled analysis sets I, II, III, and IV, respectively) (Supplementary Fig. 1). No significant asymmetry was observed between the subgroup results of the multicenter and single-institution studies (data not shown).

## Discussion

The present meta-analysis reviewed multicenter analyses and single institutional series to evaluate whether rectal cancer patients who achieved a pCR after preoperative CRT could benefit from ACT. Despite a trend toward better survival with ACT, statistical significance was not consistent in the different sets of overall pooled analyses. The stratified analysis comparing the results from single institution and multicenter studies failed to show a significant prognostic benefit. This study provides an updated perspective on the optimal postoperative strategy in cases with a marked treatment response after neoadjuvant CRT.

Based on ambiguous guidelines, patients with more advanced disease and a poorer response to CRT are potential candidates for ACT. A higher ypT and/or N stage are indicative of unfavorable tumor biology, which highlights the need for aggressive postoperative treatment to eradicate any residual tumor burden. Fietkau *et al*. failed to demonstrate any benefit of ACT in ypN0 patients (*P* = 0.993 for 3-year DFS), suggesting the need for more intensive systemic management of ypN2 patients with a poorer prognosis^12^. Another pooled analysis of the EORTC 22921 trial and an Italian trial also showed no favorable effect of ACT in patients showing a pathological response (HR of death and recurrence or death [95% CI]: 0.96 [0.75–1.23] and 0.95 [0.73–1.23], respectively)^13^. In contrast, ACT has often been recommended in down-staged patients after CRT. Based on the responsiveness to preoperative cytotoxic treatment, eradicating micrometastatic disease and further beneficial effects arising from ACT can be expected. A meta-analysis of relevant trials showed that the 5-year OS was significantly higher following ACT in patients with ypT0-2N0M0 (i.e., down-staged) (odds ratio 0.57, 95% CI 0.38–0.85)^10^. In a pooled analysis including individual patient data from the I-CNR-RT, PROCTOR-SCRIPT, EORTC 22921, and CHRONICLE trials, unlike the ypII subgroup (HR [95% CI] of OS, 0.87 [0.65–1.18]), poor responders in the ypIII stage did not show outcomes that favored the use of ACT (HR [95% CI] of OS, 1.09 [0.86–1.38])^32^.

A pCR, characterized as maximal down-staging that can be achieved by CRT, yields a high 5-year survival rate of ~85–90%^33–35^. The lack of prospective studies to evaluate the effect of ACT is because a pCR is only achieved in ~10–20% of all rectal cancer patients^14,15^. Additionally, treatment adherence to ACT in this subpopulation is mostly poor given the possibility of treatment-related toxicity, financial burden, and a patient or clinician’s preference for less aggressive treatment^36^. In this clinical setting, a meta-analytic approach with a larger number of patients can help detect a small or absent treatment effect.

The results from this meta-analysis suggest there is no clear evidence to support survival benefits of ACT for pCR patients. The trend toward a favorable prognosis with ACT was mainly based on NCDB studies, and other exploratory analyses excluding the population-based data failed to obtain any reproducible results. Although a recent meta-analysis from a Chinese group suggested the potential survival benefit of ACT in pCR patients^37^, only one NCDB study of Polanco *et al*.^29^. was included without considering the other three NCDB studies^28,30,31^. Specifically, the propensity score matching method used by Polanco *et al*. may have produced additional selection bias due to limited patient information, such as underlying morbidity or immediate mortality, within a few months after completion of treatment. Chang also noted that the survival benefit of a certain treatment can be overestimated in the statistical setting of NCDB data^38^. Therefore, we believe that the trend toward better survival in the ACT group, with the underlying predominance of one NCDB-based study, should be interpreted with caution.

The study of Maas *et al*. analyzing individual-level data of pCR patients from 13 institutions could not prove any benefits of ACT in terms of survival or tumor recurrence (HR 0.94, 95% CI 0.53–1.69 for DFS)^27^. Multicenter studies from Taiwan, Italy/Spain, and Australia^24–26^ failed to identify a survival difference, as did a recent multi-institutional study from Korea (n = 118 for pCR, from personal communication)^39^. The I-CNR-RT trial could not analyze outcomes of pCR patients due to the low incidence of mortality events^2^. To date, there is little randomized evidence to demonstrate the survival benefit of ACT in this patient population.

To assess the clinical value of ACT, potential adverse effects also need to be considered. Among the 13 studies, only two reported ACT-related complication events^20,26^. In the study of Tay *et al*., 13% of study population stopped ACT due to significant toxicities, such as diarrhea, nausea, and vomiting^26^. Time to closure of ileostomy was longer in patients with ACT, suggesting inferior quality of life and related medical morbidities^20^. Although life-threatening complication is not common (~1–2%), grade 3–4 acute toxicities were reported in more than one third of patients after the use of multi-agent ACT regimens^40,41^. Regarding the inconsistent survival outcomes in the present pooled analyses including four different NCDB-based studies, it is questionable whether the therapeutic benefit of ACT is sufficiently expected to risk treatment-related toxicities and quality-of-life problems in patients with a marked response to CRT.

This study had several limitations. First, all of the included studies were observational and inevitably suffer from confounders, such as selection bias and heterogeneity in sample characteristics and treatments^42^. Of the included studies, the method of Tierney *et al*.^19^ to extract HR and 95% CI data was used in 5 studies^20,22,23,25,26^. The differences between the data indirectly extracted from survival curves and the original data can induce additional bias. Due to insufficient data, the effect of ACT on DFS or treatment-related toxicities was not evaluated in the present meta-analysis. Most of the studies included herein did not account for the presence of underlying comorbid illnesses or postoperative complications. Use of 5-fluorouracil, 5-fluorouracil/leucovorin (FL), and capecitabine chemotherapy was reported in most studies, but it remains unclear whether the current results are also applicable to other regimens, such as multi-agent folinic acid/fluorouracil/oxaliplatin (FOLFOX) and folinic acid/fluorouracil/irinotecan (FOLFIRI). Nevertheless, this large-scale meta-analysis provides clinically useful insights into the prognostic role of ACT in this small-sized population who achieved a pCR.

This meta-analysis could not warrant the survival benefits of ACT in patients who achieved a pCR, suggesting that routine use of ACT should not be recommended in this subset of patients with rectal cancer. Further pooled analysis of individual patient data from existing randomized trials is needed to establish guidelines for ACT in conjunction with contemporary neoadjuvant treatments.

## Supplementary information

Appendix 1, Appendix 2, Supplementary Figure 1

## References

[1] Siegel RL, Miller KD, Jemal A (2018). Cancer statistics, 2018. CA Cancer J Clin.

[2] Sainato A (2014). No benefit of adjuvant Fluorouracil Leucovorin chemotherapy after neoadjuvant chemoradiotherapy in locally advanced cancer of the rectum (LARC): Long term results of a randomized trial (I-CNR-RT). Radiother Oncol.

[3] Bosset JF (2014). Fluorouracil-based adjuvant chemotherapy after preoperative chemoradiotherapy in rectal cancer: long-term results of the EORTC 22921 randomised study. Lancet Oncol.

[4] Sauer R (2004). Preoperative versus postoperative chemoradiotherapy for rectal cancer. N Engl J Med.

[5] National Comprehensive Cancer Network. *Rectal Cancer - Version 2.2018*, https://www.nccn.org/professionals/physician_gls/pdf/rectal.pdf.

[6] Glynne-Jones, R. *et al*. Rectal cancer: ESMO Clinical Practice Guidelines for diagnosis, treatment and follow-up. *Ann Oncol* (2018). 10.1093/annonc/mdy161 29741565

[7] Gray R (2007). Adjuvant chemotherapy versus observation in patients with colorectal cancer: a randomised study. Lancet.

[8] Collette L (2007). Patients with curative resection of cT3-4 rectal cancer after preoperative radiotherapy or radiochemotherapy: does anybody benefit from adjuvant fluorouracil-based chemotherapy? A trial of the European Organisation for Research and Treatment of Cancer Radiation Oncology Group. J Clin Oncol.

[9] Janjan NA (2001). Improved overall survival among responders to preoperative chemoradiation for locally advanced rectal cancer. Am J Clin Oncol.

[10] Petrelli F, Coinu A, Lonati V, Barni S (2015). A systematic review and meta-analysis of adjuvant chemotherapy after neoadjuvant treatment and surgery for rectal cancer. Int J Colorectal Dis.

[11] Chan AK (2000). Preoperative chemotherapy and pelvic radiation for tethered or fixed rectal cancer: a phase II dose escalation study. Int J Radiat Oncol Biol Phys.

[12] Fietkau R (2006). Postoperative chemotherapy may not be necessary for patients with ypN0-category after neoadjuvant chemoradiotherapy of rectal cancer. Dis Colon Rectum.

[13] Bujko K, Glimelius B, Valentini V, Michalski W, Spalek M (2015). Postoperative chemotherapy in patients with rectal cancer receiving preoperative radio(chemo)therapy: A meta-analysis of randomized trials comparing surgery +/− a fluoropyrimidine and surgery + a fluoropyrimidine +/− oxaliplatin. Eur J Surg Oncol.

[14] Lorimer PD (2017). Pathologic complete response rates after neoadjuvant treatment in rectal cancer: an analysis of the National Cancer Database. Ann Surg Oncol.

[15] Lefevre JH (2016). Effect of interval (7 or 11 weeks) between neoadjuvant radiochemotherapy and surgery on complete pathologic response in rectal cancer: a multicenter, randomized, controlled trial (GRECCAR-6). J Clin Oncol.

[16] Stroup DF (2000). Meta-analysis of observational studies in epidemiology: a proposal for reporting. Meta-analysis Of Observational Studies in Epidemiology (MOOSE) group. JAMA.

[17] Song Changhoon, Chung Joo-Hyun, Kang Sung-Bum, Kim Duck-Woo, Oh Heung-Kwon, Lee Hye Seung, Kim Jin Won, Lee Keun-Wook, Kim Jee Hyun, Kim Jae-Sung (2018). Impact of Tumor Regression Grade as a Major Prognostic Factor in Locally Advanced Rectal Cancer after Neoadjuvant Chemoradiotherapy: A Proposal for a Modified Staging System. Cancers.

[18] Kim SY (2013). Testing a tool for assessing the risk of bias for nonrandomized studies showed moderate reliability and promising validity. J Clin Epidemiol.

[19] Tierney JF, Stewart LA, Ghersi D, Burdett S, Sydes MR (2007). Practical methods for incorporating summary time-to-event data into meta-analysis. Trials.

[20] Gamaleldin M (2017). Is routine use of adjuvant chemotherapy for rectal cancer with complete pathological response justified?. Am J Surg.

[21] Kim CG (2017). Role of adjuvant chemotherapy in locally advanced rectal cancer with ypT0-3N0 after preoperative chemoradiation therapy and surgery. BMC Cancer.

[22] Lichthardt S (2017). Impact of adjuvant chemotherapy after neoadjuvant radio- or radiochemotherapy for patients with locally advanced rectal cancer. J Cancer Res Clin Oncol.

[23] Geva R (2014). Is there a role for adjuvant chemotherapy in pathological complete response rectal cancer tumors following neoadjuvant chemoradiotherapy?. J Cancer Res Clin Oncol.

[24] Kuan FC (2017). The survival impact of delayed surgery and adjuvant chemotherapy on stage II/III rectal cancer with pathological complete response after neoadjuvant chemoradiation. Int J Cancer.

[25] Lorenzon L (2017). Long-term outcomes in ypT0 rectal cancers: An international multi-centric investigation on behalf of Italian Society of Surgical Oncology Young Board (YSICO). Eur J Surg Oncol.

[26] Tay RY (2017). Survival impact of adjuvant chemotherapy for resected locally advanced rectal adenocarcinoma. Clin Colorectal Cancer.

[27] Maas M (2015). Adjuvant chemotherapy in rectal cancer: defining subgroups who may benefit after neoadjuvant chemoradiation and resection: a pooled analysis of 3,313 patients. Int J Cancer.

[28] Dossa F (2018). Association between adjuvant chemotherapy and overall survival in patients with rectal cancer and pathological complete response after neoadjuvant chemotherapy and resection. JAMA Oncol.

[29] Polanco PM, Mokdad AA, Zhu H, Choti MA, Huerta S (2018). Association of adjuvant chemotherapy with overall survival in patients with rectal cancer and pathologic complete response following neoadjuvant chemotherapy and resection. JAMA Oncol.

[30] Xu Z (2017). Poor compliance with adjuvant chemotherapy use associated with poorer survival in patients with rectal cancer: An NCDB analysis. Cancer.

[31] Shahab D (2017). Adjuvant chemotherapy is associated with improved overall survival in locally advanced rectal cancer after achievement of a pathologic complete response to chemoradiation. Clin Colorectal Cancer.

[32] Breugom AJ (2015). Adjuvant chemotherapy after preoperative (chemo)radiotherapy and surgery for patients with rectal cancer: a systematic review and meta-analysis of individual patient data. Lancet Oncol.

[33] Das P (2006). Clinical and pathologic predictors of locoregional recurrence, distant metastasis, and overall survival in patients treated with chemoradiation and mesorectal excision for rectal cancer. Am J Clin Oncol.

[34] Park IJ (2012). Neoadjuvant treatment response as an early response indicator for patients with rectal cancer. J Clin Oncol.

[35] Capirci C (2008). Prognostic value of pathologic complete response after neoadjuvant therapy in locally advanced rectal cancer: long-term analysis of 566 ypCR patients. Int J Radiat Oncol Biol Phys.

[36] Garcia-Albeniz X (2014). Adjuvant therapy sparing in rectal cancer achieving complete response after chemoradiation. World J Gastroenterol.

[37] Ma, B. *et al*. Is adjuvant chemotherapy necessary for locally advanced rectal cancer patients with pathological complete response after neoadjuvant chemoradiotherapy and radical surgery? A systematic review and meta-analysis. *Int J Colorectal Dis* (2018). 10.1007/s00384-018-3181-9 30368569

[38] Chang GJ (2018). Is there validity in propensity score-matched estimates of adjuvant chemotherapy effects for patients with rectal cancer?. JAMA Oncol.

[39] Chung MJ (2019). Adjuvant chemotherapy in rectal cancer patients treated with preoperative chemoradiation and total mesorectal excision: a multicenter and retrospective propensity-score matching study. Int J Radiat Oncol Biol Phys.

[40] Glynne-Jones R (2014). Chronicle: results of a randomised phase III trial in locally advanced rectal cancer after neoadjuvant chemoradiation randomising postoperative adjuvant capecitabine plus oxaliplatin (XELOX) versus control. Ann Oncol.

[41] Hong YS (2014). Oxaliplatin, fluorouracil, and leucovorin versus fluorouracil and leucovorin as adjuvant chemotherapy for locally advanced rectal cancer after preoperative chemoradiotherapy (ADORE): an open-label, multicentre, phase 2, randomised controlled trial. Lancet Oncol.

[42] Hammer GP, du Prel JB, Blettner M (2009). Avoiding bias in observational studies: part 8 in a series of articles on evaluation of scientific publications. Dtsch Arztebl Int.

