# Supplementary material for: Adjuvant chemotherapy in rectal cancer patients who achieved a pathological complete response after preoperative chemoradiotherapy: a systematic review and meta-analysis
Source: Sci Rep. 2019 Jul 10;9:10008. doi: 10.1038/s41598-019-46457-5 (PMC6620266; doi:10.1038/s41598-019-46457-5)
Supplement: Supplementary file 1 — Appendix 1, Appendix 2, Supplementary Figure 1 [file 41598_2019_46457_MOESM1_ESM.docx]

**Adjuvant chemotherapy in rectal cancer patients who achieved a pathological complete response after preoperative chemoradiotherapy: a systematic review and meta-analysis**

Yu Jin Lim, Youngkyong Kim, and Moonkyoo Kong^*^

^1^Department of Radiation Oncology, Kyung Hee University Medical Center, Kyung Hee University School of Medicine, Seoul, Republic of Korea

**^*^Correspondence:** Moonkyoo Kong

Department of Radiation Oncology, Kyung Hee University Medical Center, Kyung Hee University School of Medicine, 23 Kyungheedae-ro, Dongdaemoon-gu, Seoul, 02447

Telephone: +82-2-958-8661

Fax: +82-2-958-9469

E-mail: [kongmoonkyoo@khu.ac.kr](mailto:kongmoonkyoo@khu.ac.kr)

**Appendix 1.** The Meta-analysis of Observational Studies in Epidemiology (MOOSE) checklist

| **Criteria** | **The part or page number of the present meta-analysis in relation to the corresponding criteria** |
| --- | --- |
| **Reporting of background should include** |  |
| Problem definition | Introduction |
| Hypothesis statement | Introduction |
| Description of study outcome(s) | Introduction  Methods and materials – Study selection  Methods and materials – Statistical analysis |
| Type of exposure or intervention used | Methods and materials – Study selection |
| Type of study designs used | Methods and materials – Literature search strategy  Methods and materials – Study selection  Figure 1 |
| Study population | Methods and materials – Study selection |
| **Reporting of search strategy should include** |  |
| Qualifications of searchers | Methods and materials – Study selection |
| Search strategy, including time period included in the synthesis and keywords | Methods and materials – Literature search strategy |
| Effort to include all available studies, including contact with authors | Methods and materials – Study selection & Data extraction |
| Databases and registries searched | Methods and materials – Literature search strategy |
| Search software used, name and version, including special features used | Methods and materials – Statistical analysis |
| Use of hand searching | Methods and materials – Literature search strategy |
| List of citations located and those excluded, including justification | Figure 1  Others excluded in the selection process are available upon request. |
| Method of addressing articles published in languages other than English | Supplementary material – Appendix 2 includes the details of searching strategy including the language limitation. |
| Method of handling abstracts and unpublished studies | Methods and materials – Study selection & Data extraction |
| Description of any contact with authors | Methods and materials – Data extraction |
| **Reporting of methods should include** |  |
| Description of relevance or appropriateness of studies assembled for assessing the hypothesis to be tested | Methods and materials – Literature search strategy & Study selection |
| Rationale for the selection and coding of data | Methods and materials – Statistical analysis  Relevant files of data extraction are available on request. |
| Documentation of how data were classified and coded | Methods and materials – Statistical analysis |
| Assessment of confounding | Results – Study characteristics  Table 2 |
| Assessment of study quality, including blinding of quality assessors; stratification or regression on possible predictors of study results | Results – Study characteristics  Table 2 |
| Assessment of heterogeneity | Results – Comparison of OS with and without ACT  Results – Subgroup analyses |
| Description of statistical methods | Methods and materials – Statistical analysis |
| Provision of appropriate tables and graphics | Table 1, Table 2, Figure 1, Figure 2, Figure 3  Supplementary Materials |
| **Reporting of results should include** |  |
| Graphic summarizing individual study estimates and overall estimate | Figure 2  Supplementary Figure 1 |
| Table giving descriptive information for each study included | Table 1 |
| Results of sensitivity testing | Results – Subgroup analyses  Supplementary Figure 1 |
| Indication of statistical uncertainty of findings | 95% confidence interval values were presented with I^2^ values for the pooled analyses. |
| **Reporting of discussion should include** |  |
| Quantitative assessment of bias | Methods and materials – Statistical analysis  Results – Publication bias |
| Justification for exclusion | Methods and materials – Study selection  Figure 1 represents the exclusion process. |
| Assessment of quality of included studies | Results – Study characteristics  Table 2  Discussion (addressing the study limitations) |
| **Reporting of conclusions should include** |  |
| Consideration of alterative explanations for observed results | Discussion (addressing the study limitations) |
| Generalization of the conclusions | Conclusions |
| Guidelines for future research | Conclusions |
| Disclosure of funding source | Acknowledgements |

**Appendix 2.** Searching terms and strategy

**1. EMBASE**

#1. (rectum:ab,ti OR rectal:ab,ti) AND [1990-2018]/py

#2. (cancer:ab,ti OR carcinoma:ab,ti) AND [1990-2018]/py

#3. (postop*:ab,ti OR adjuvant:ab,ti) AND [1990-2018]/py

#4. chemotherapy:ab,ti AND [1990-2018]/py

#5. (preop*:ab,ti OR neoadjuvant:ab,ti) AND [1990-2018]py

#6. (chemoradiotherapy:ab,ti OR chemoradiation:ab,ti OR 'chemo radiation':ab,ti OR 'radio chemotherapy':ab,ti OR 'chemo radiotherapy':ab,ti OR radiochemotherapy) AND [1990-2018]/py

#7. #1 AND #2

#8. #3 AND #4

#9. #5 AND #6

#10. #7 AND #8 AND #9

#11. #10 NOT ([editorial]/lim OR [erratum]/lim OR [letter]/lim OR [review]/lim) AND [english]/lim

**2. PubMed**

#1. Search rectal[Title/Abstract] OR rectum[Title/Abstract] Filters: Publication date from 1990/01/01 to 2018/09/30; English

#2. Search cancer[Title/Abstract] OR carcinoma[Title/Abstract] Filters: Publication date from 1990/01/01 to 2018/09/30; English

#3. Search postop* or adjuvant Filters: Publication date from 1990/01/01 to 2018/09/30; English

#4. Search chemotherapy[Title/Abstract] Filters: Publication date from 1990/01/01 to 2018/09/30; English

#5. Search preop* or neoadjuvant Filters: Publication date from 1990/01/01 to 2018/09/30; English

#6. Search chemoradiation[Title/Abstract] OR chemoradioth* OR chemo-radiotherapy[Title/Abstract] OR radiochemotherapy[Title/Abstract] OR chemo-radiation[Title/Abstract] OR radio-chemotherapy[Title/Abstract] OR radiochemoth* Filters: Publication date from 1990/01/01 to 2018/09/30; English

#7. Search #1 AND #2 Filters: Publication date from 1990/01/01 to 2018/09/30; English

#8. Search #3 AND #4 Filters: Publication date from 1990/01/01 to 2018/09/30; English

#9. Search #5 AND #6 Filters: Publication date from 1990/01/01 to 2018/09/30; English

#10. Search #7 AND #8 AND #9 Filters: Publication date from 1990/01/01 to 2018/09/30; English

**3. Web of Science**

#1. (TS=(rectum or rectal)) *AND* LANGUAGE: (English) *AND* DOCUMENT TYPES: (Article)

*Indexes=SCI-EXPANDED, SSCI, A&HCI, ESCI Timespan=1990-2018*

#2. (TS=(cancer or carcinoma)) *AND* LANGUAGE: (English) *AND* DOCUMENT TYPES: (Article)

*Indexes=SCI-EXPANDED, SSCI, A&HCI, ESCI Timespan=1990-2018*

#3. (TS=(chemotherapy)) *AND* LANGUAGE: (English) *AND* DOCUMENT TYPES: (Article)

*Indexes=SCI-EXPANDED, SSCI, A&HCI, ESCI Timespan=1990-2018*

#4. (TS=(postop* or adjuvant)) *AND* LANGUAGE: (English) *AND* DOCUMENT TYPES: (Article)

*Indexes=SCI-EXPANDED, SSCI, A&HCI, ESCI Timespan=1990-2018*

#5. (TS=(chemoradiotherapy OR chemoradiation OR chemo-radiation OR radio-chemotherapy OR chemo-radiotherapy OR radiochemotherapy)) *AND* LANGUAGE: (English) *AND* DOCUMENT TYPES: (Article)

*Indexes=SCI-EXPANDED, SSCI, A&HCI, ESCI Timespan=1990-2018*

#6. (TS=(preop* or neoadjuvant)) *AND* LANGUAGE: (English) *AND* DOCUMENT TYPES: (Article)

*Indexes=SCI-EXPANDED, SSCI, A&HCI, ESCI Timespan=1990-2018*

#7. ((#1 and #2) and (#3 and #4) and (#5 and #6)) *AND* LANGUAGE: (English) *AND* DOCUMENT TYPES: (Article)

*Indexes=SCI-EXPANDED, SSCI, A&HCI, ESCI Timespan=1990-2018*

**4. Ovid**

#1. ((rectum or rectal) and (cancer or carcinoma)).ab.

#2. ((postop* or adjuvant) and (chemotherapy)).ab.

#3. ((preop* or neoadjuvant) and (chemoradiotherapy or chemoradiation or chemo-radiation or radio-chemotherapy or chemo-radiotherapy or radiochemotherapy)).ab.

#4. #1 and #2 and #3

*⇒ Filters: Publication year 1990-2018; Language English*

**5. Cochrane Library**

#1. “rectum”:ti,ab,kw or “rectal”:ti,ab,kw

#2. “carcinoma”:ti,ab or ”cancer”:ti,ab

#3. “chemotherapy”:ti,ab

#4. “postoperative”:ti,ab or “adjuvant”:ti,ab or “postoperation”:ti,ab

#5. “chemoradiotherapy”:ti,ab or “chemoradiation”:ti,ab or “chemo-radiation”:ti,ab or “radio-chemotherapy”:ti,ab or “chemo-radiotherapy”:ti,ab or “radiochemotherapy”:ti,ab

#6. “preoperative”:ti,ab or “neoadjuvant”:ti,ab or “preoperation”:ti,ab

#7. (#1 and #2) and (#3 and #4) and (#5 and #6)

*⇒ Filters: Publication year 1990-2018*


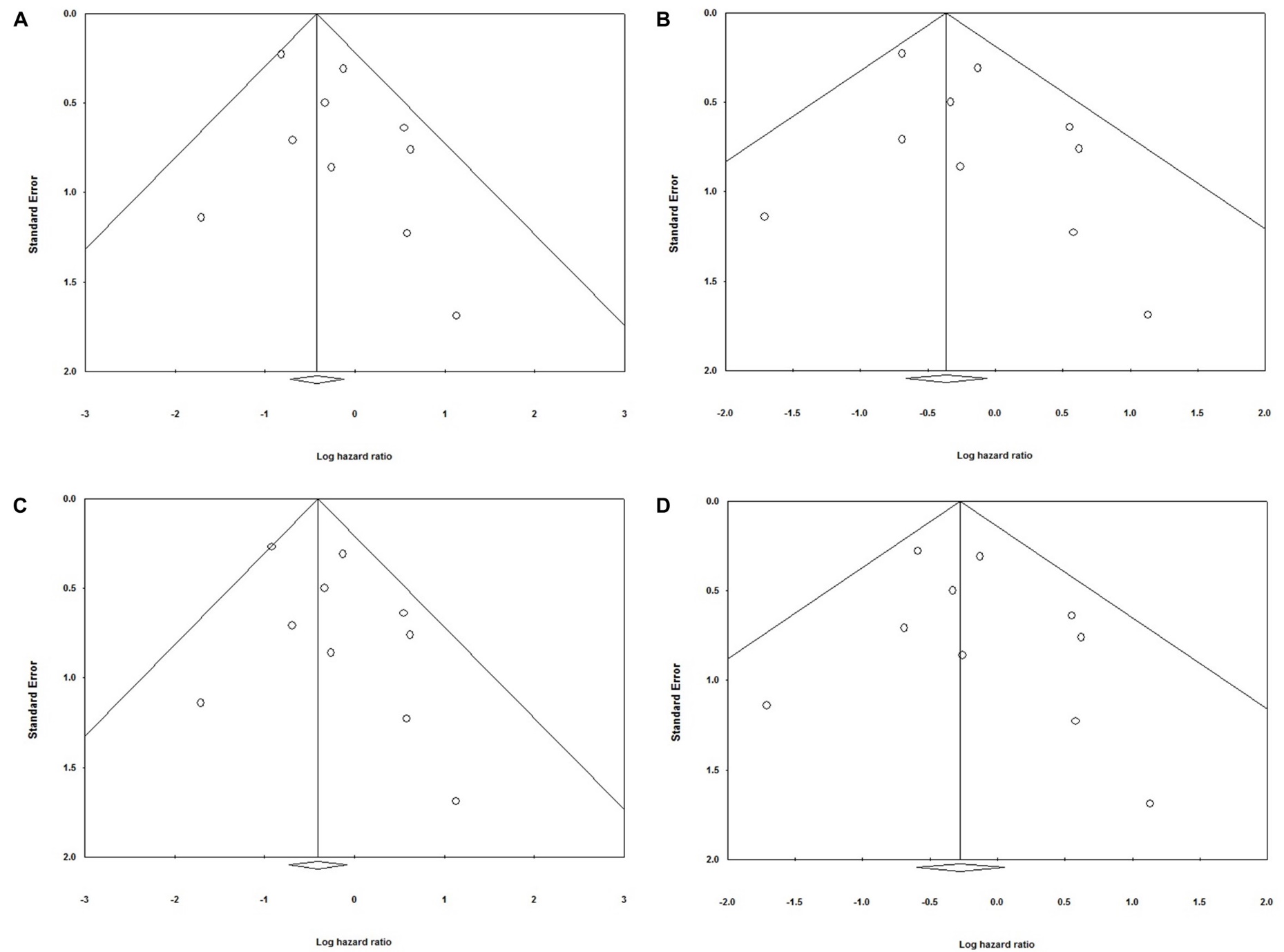


**Supplementary Figure 1.** Funnel plots showing log hazard ratios and standard errors: pooled-analysis dataset (A) I, (B) II, (C) III, and (D) IV, including the study of Dossa et al., Polanco et al., Xu et al., and Shahab et al., respectively.
